# In vitro anticancer activity of *Eclipta alba* whole plant extract on colon cancer cell HCT-116

**DOI:** 10.1186/s12906-020-03118-9

**Published:** 2020-11-23

**Authors:** Vinod kumar Nelson, Nalini Kanta Sahoo, Madhusmita Sahu, Hari hara Sudhan, Chitikela P. Pullaiah, Kanuri Sai Muralikrishna

**Affiliations:** 1 Raghavendra Institute of Pharmaceutical Education & Research (Autonomous), Anantapuramu, Andhra Pradesh 515721 India; 2 Marri Laxman Reddy Institute of Pharmacy, Medchal, Dundigal, Telangana 500043 India; 3 Department of Pharmacology, Siddha Central Research Institute, Chennai, Tamil Nadu 60016 India

**Keywords:** Colon cancer, *Eclipta alba*, Specificity, Anticancer, Nontoxic, Normal cells

## Abstract

**Backgrounds:**

Colon cancer is the third most deadly and one of the most diagnosed diseases in the world. Although routine screening and early detection during last decades has improved the survival, colon cancer still claims hundreds of thousands lives each year worldwide. Surgery and chemotherapy is mainstay of current treatment, nevertheless toxicity associated with this treatment underscores the urgency of demand of a better therapeutics. Close to 50% of current chemotherapeutic drugs are direct or indirect descendants compounds isolated from medicinal plants, which indicate plants are great potential sources of novel therapeutics. In our literature review we found *Eclipta alba* to posses many pharmacological activities, including those with anticancer potentials. However, no study on anticancer activity of this kind has been reported.

**Methods:**

Phytochemicals were extracted by maceration method from shade dried whole plant of *Eclipta alba* using methanol as a solvent. The anticancer effect of extract was investigated on various cancer cell lines like human colorectal carcinoma (HCT-116), human prostate cancer (PC-3), Michigan cancer foundation-breast cancer (MCF-7) and renal cell carcinoma (RCC-45). We have also studied the effects on normal human embryonic lung fibroblast cell (WI-38) using MTT (methyl thiazoldiphenyltetrazolium bromide) assay, clonogenic (colony formation) and migration assay. Finally obtained results were analyzed using ANNOVA and Dunnett’s test.

**Results:**

Results obtained from MTT assay revealed that the methanolic extract of *Eclipta alba* carried significant (*p* < 0.005) specificity against HCT-116 cells as compared to the other cancer cells. This extract also showed minimal or nontoxicity to WI-38 cells. Migration as well as clonogenic assays also confirmed the anticancer potential of the extract against HCT-116 cells.

**Conclusion:**

This is a unique finding of its kind because the specific anticancer effect with minimal toxicity on normal cells has not been reported on *Eclipta alba* extract. Finally this finding opens up a great possibility to develop a novel antitumor drug candidate against deadly colon cancer in the future.

## Background

Cancer is the second leading cause of death globally, and is responsible for an estimated 9.6 million deaths in 2018. Globally, about 1 in 6 deaths is due to cancer. Approximately 70% of deaths from cancer occur in low and middle-income countries. Opportunities to reduce the death rate from cancer through the discovery of new drugs are benefiting from the advances in technology and knowledge on neoplastic disease [1]. Cancer arises from the transformation of normal cells into tumor cells in a multistage process which include transition from a pre-cancerous lesion to a malignant tumor state. These changes are the result of the interaction between a person’s genetic factors with physical (ultra violet radiation), chemical (tobacco and asbestos) and biological (virus and bacteria) factors.

Colorectal cancer (CRC) or colon cancer positions as the second most lethal cancer and the third most prevalent malignant tumor worldwide. In 2018, of 1.8 million new CRC cases and 881,000 deaths were reported, which accounted for nearly 10% of new cancer cases and deaths worldwide [2]. There has been a great advancement in cancer treatment which enhanced the patient survival and quality of life. However, cancer related deaths are continuously rising [3]. Generally CRC in early stage appears as an abnormal growth on the inner walls of colon epithelium cells which can be removed by surgery if detected early. However, if the patient at earlier stage is left untreated, the cancer cells get metastasized to different sites on different organs when the cancer becomes even insensitive to chemotherapy [4].

Cancer develops as a result of deregulation of single and or multiple cellular mechanism(s) involved in, such as, cell division, and apoptosis that are required by normal growth and proliferation of healthy cells. Identifying the difference(s) in regulatory mechanism at play in cancer cell responsible for transformation, and specifically targeting that mechanism is the major task for drug development and candidate screening [5–7].

Current choices for the treatments of CRC are chemotherapy with single drug fluoropyrimidine and multiple agent regimens including oxaliplatin, irinotecan, and capecitabine. Moreover, the ideal CRC treatment is to achieve complete removal of the tumor and metastases, which mostly requires surgical interventions. In certain cases, chemotherapy or radiotherapy might be applied before or after surgery as neoadjuvant or adjuvant treatment to maximally reduce and stabilize the tumor [8].

However, the existing treatment options for CRC are complex and associated with toxic side effects. Therefore, the researchers are in search of novel drug candidates with minimal toxicity towards the normal/noncancerous cells. Due to unique structural nature, vast diversity in their chemical properties and minimal toxicity, secondary metabolites available in medicinal plants are considered an attractive target for screening of novel drug candidates against the dreadful diseases such as cancer [9].

*Eclipta alba* (L.) Hassk (synonym *Eclipta prostrata*) is an annual herbaceous plant, erect or prostrate, belonging to the *Asteraceae* family. It is also known as Bhringaraj in Ayurveda which has been in use for treating different ailments especially related to the liver and hair [10]. The herb has been known as antimycotoxic, analgesic, antibacterial, antihepatotoxic, anti-hemorrhagic, antihyperglycemic, antioxidant and immunomodulatory and rejuvenating properties. Coumestan, wedelolactone, desmethylwedelolactone as well as β-sitosterol present in this plant are known for their antihepatotoxic, antioxidant and anticancer activities [11].

Wedelolactone was shown to induce apoptosis via activation of c-Jun N-terminal kinase (JNK) and caspase-3., Oleanolic acid was shown to be cytotoxic to cancer cell through inducing cell cycle arrest and apoptosis. Eclalbasaponins and β-sitosterol was shown to kill cancer cells by apoptosis through induction of oxidative stress, and DNA damage [12, 13].

The present study mainly aims to evaluate anticancer potential of methanolic extract of *Eclipta alba* (L.) on various types of cancer cells HCT-116, PC-3, RCC-45, and MCF-7 through employing various assay methods such as morphological studies, cell viability, wound healing and colony formation assays. To evaluate general toxicity normal cell WI-38 was also included in the assays.

## Methods

### Cell lines and cell culture preparation

HCT-116, HT-29, WI-38, MCF-7, PC-3 and RCC-45 cell lines were obtained from NCCS Pune. HCT-116, and WI-38 cells were cultured in Dulbecco’s Modified Eagle’s Medium (DMEM) and MCF-7, PC-3, and RCC-45 cells were cultured in Roswell Park Memorial Institute (RPMI) medium added with 10% FBS, 1% L-Glutamine, 0.1 mM (milli Molar) nonessential amino acid, and 100 U/ml penicillin/streptomycin. Cells were incubated in humidified incubator at 37 °C with 5% CO_2_.

### Collection of the plant

The whole plant of *Eclipta alba* for the proposed study was collected in the month of January, 2019 from the fields of Tirupati, India and authentication was confirmed by Dr. N. Savithramma, Professor of Botany. S. V. University, Tirupati, India, and the specimen voucher number 695 in the form of herbarium were deposited in the department.

### Preparation of methanolic extract of *Eclipta alba*

The plant was shade dried and grounded into fine powder. Approximately 1000 g of plant powder was kept for extraction with about 1500 ml methanol for 3 days with frequent shaking to extract polar constituents like phytosterols, polyphenols, flavonoids, terpenoids and different coumarin derivatives of the plant. Later the extract was collected by filtration using a whattman filter paper (Merck). This process was repeated for 3 times for nearly complete extraction of the all soluble constituents. The extract (pooled together all three batches of filtrates) was finally concentrated by evaporating the solvent with a rotary vacuum evaporator and kept at 4 °C till use. All chemical used in the present study were of analytical grade.

### Sample preparation and treatment

Methanol extracts’ concentrate of *Eclipta alba* weighed exactly 100 mg was dissolved in 1 ml dimethyl sulfoxide (DMSO) and stored in − 20 °C until use. Cells were grown to ~ 70% confluence in a 24-well plate and treated with a range of concentrations such as 0-, 50-, 100-, 200-, 400- and 500 μg/ml of extract. The final concentration of the DMSO in the treatment was maintained at less than 0.25%. Each and every experiment was repeated for at least 3 times to acquire the data.

### Cell viability assay

Cells were grown in respective media in 96-well plates to ~ 70% confluence and the cells were treated with different concentrations of extract along with the vehicle/DMSO control for about 24 h. Next, the media was aspirated to wash the cells with phosphate buffer Saline (PBS). MTT solution at 0.5 mg/ml was added to each well and the plate was incubated at 37 °C for 4 h in the dark following which the MTT solution was replaced with 200 μl of DMSO. The plate was shaken at 150 rpm for 5 min and the optical density was measured at 490 nm by using a plate reader (ELx 800; Biotek, Winooski, VT, USA). The experiment was repeated for at least 3 times before the data was calculated to plot a graph [14–16].

### Morphology study

Cells were plated in 24-well plates and treated with DMSO or extract (at IC-50 concentration) for 24 h and after treatment period the image was capture by using phase contrast microscopy.

### Clonogenic assay

Cells were grown to ~ 80% confluency and treated with specific concentration of vehicle or extract for 24 h. After 24 h cells were trypsinized and seeded at about 10 × 10^3^ cells in each well of a 6-well plate and allowed to grow for 14 days. Next, the cells were washed with PBS and incubated with 0.5% crystal violet solution (containing 3.7% formaldehyde) for 30 min. Then the crystal violet was washed off with running tap water and plates were allowed to dry at room temperature. The image was captured and density was measured using Gel Doc XR + (Bio-Rad) [17].

### Wound healing assay

Cells were seeded in 6-well plates and allowed to grow up to 80–90% confluency when a uniform scratch/wound in each well was made with a 10 μl pipette tip. The cells were washed with sterile PBS to remove the debris to treat with different concentrations of extract or vehicle for 24 h or 48 h following which images of the cells were obtained using an inverted microscope. All experiments were repeated three times to record the data [18].

### Statistical analysis

Results were expressed as mean ± standard error mean multiple comparisons of the significant analysis of variance (ANOVA) followed by the Dunnett’s test as post parametric test using computer based fitting program (Prism graph pad 5.0). A ‘p’ value of < 0.05 was considered as statistically significant.

## Results

### *Eclipta alba* methanol extract exhibits targeted anticancer effect against colon cancer cell line

**Fig. 1:**
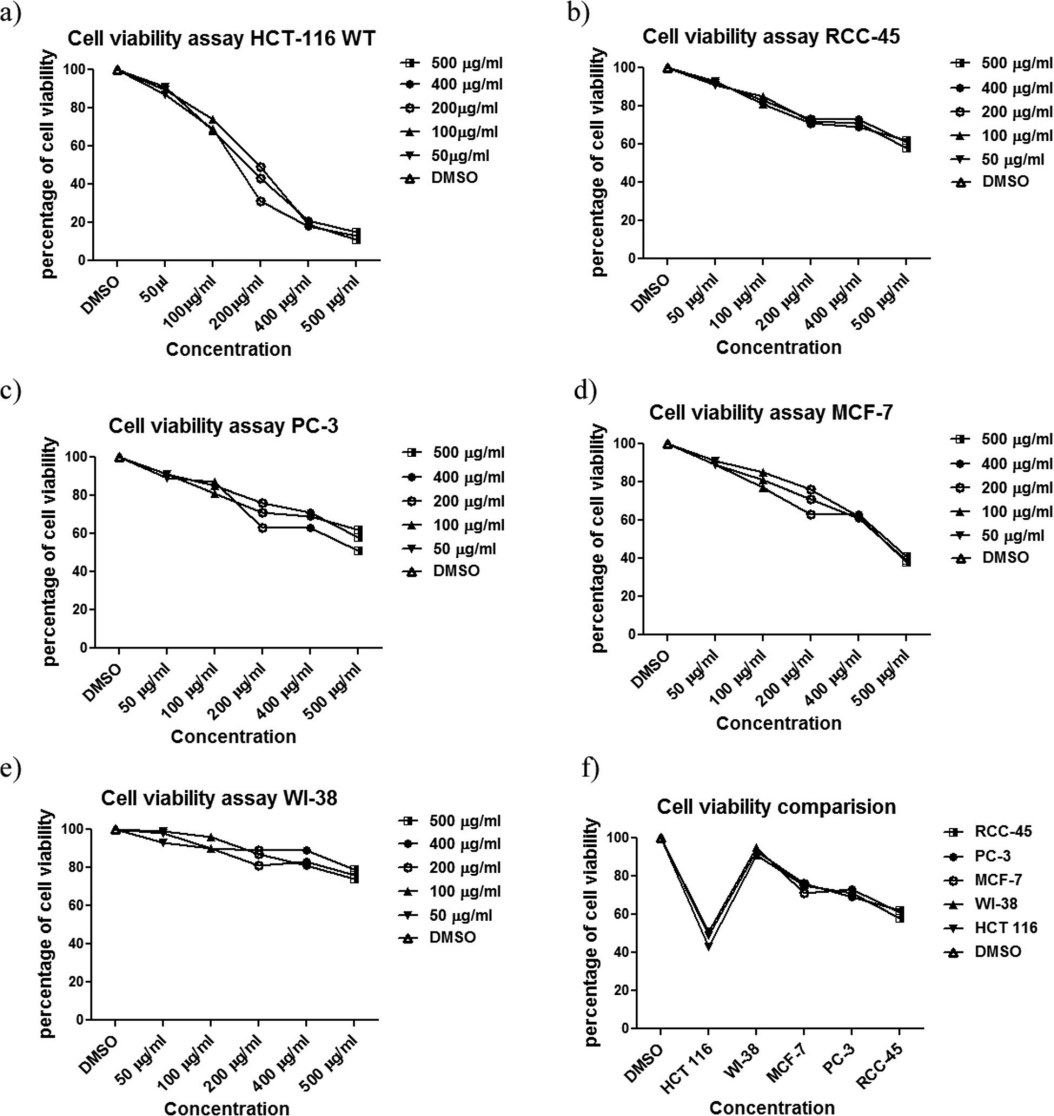
The MTT assay results show *Eclipta alba* methanol extract (EAME) exhibits specific growth inhibition in colon cancer cells (graph **a**) as compared to other selected cancer cells (graph panel **b**, **c** and **d**), the results also reveals extract shows negligible toxicity to the normal cell (graph **e**). Right bottom graph shows relative sensitivities of the cells that were compared by plotting their IC-50 values

**Fig. 2:**
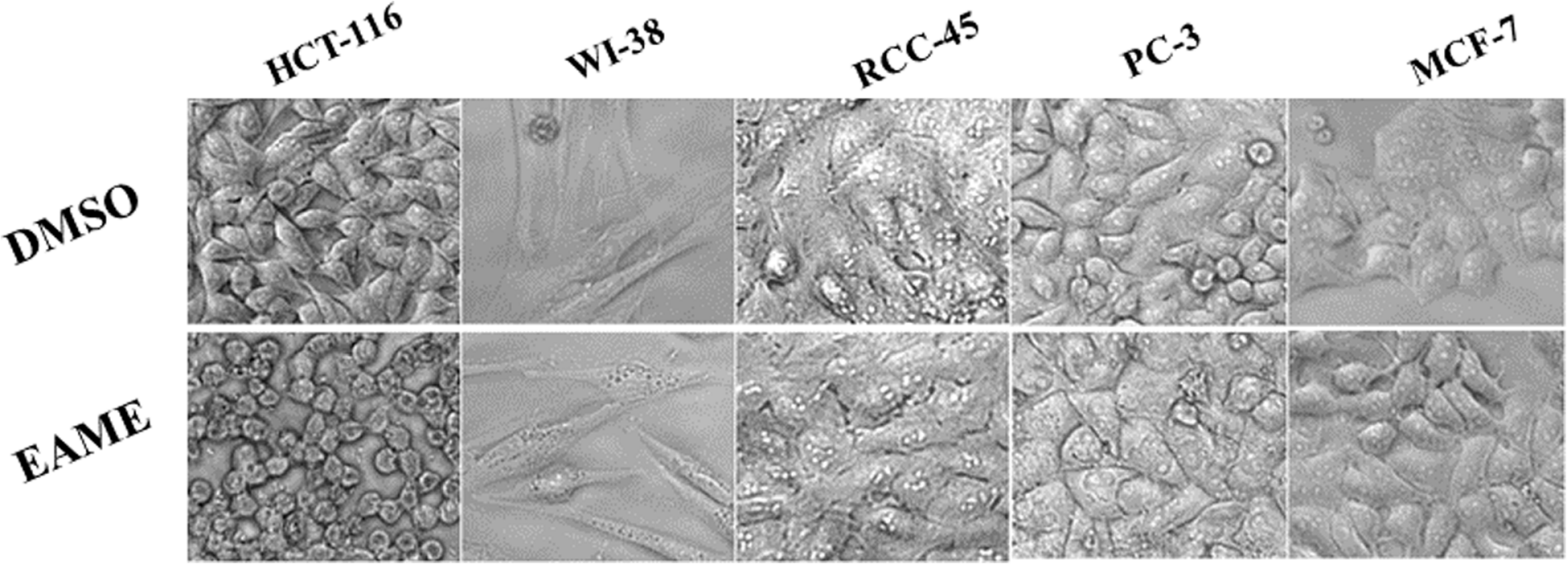
The Phase contrast image shows specific and significant morphological change in HCT-116 cells as compared to other cancer cells (RCC-45, PC-3 and MCF-7) and also normal cell (WI-38) as treated with *Eclipta alba* methanol extract at their IC-50 concentrations

Anticancer effect of methanolic extract of *Echlipta alba* was estimated by MTT assay and morphological studies. The results of the MTT assay and morphological study shown in Figs. 1 & 2, revealed specific anticancer activity of the extract towards colon cancer cell line HCT-116 compared to breast cancer cell line (MCF-7), prostate cancer cell line (PC-3) and renal cancer cell line (RCC-45). The plant extract exhibited a significant (*p* < 0.05) cytotoxicity towards HCT-116 cells in dose-dependent manner as compared to other cancer cells (PC-3, MCF-7 and RCC-45) while the normal cells WI-38 showed much less sensitivity. The IC-50 for HCT-116 cells was calculated to be 179 ± 0.81 μg/ml, whereas IC-50 values of MCF-7, PC-3 and RCC-45 cells were found to be 400 ± 1.01, 470 ± 1.04 and 498 ± 1.90 μg/ml, respectively (*p* < 0.05). Relatively much higher IC-50 (576 ± 0.98 μg/ml) of WI-38 supported relative nontoxicity/safety of the extract to the normal cells. Morphological studies, migration assay and clonogenic assays were carried out at 100 and 200 μg/ml concentrations of the extract. The concentrations were chosen based on IC-50 concentration of HCT-116 cells (179 ± 0.81 μg/ml). Morphological study also shows significant effect on the morphology of HCT-116 cells treated with the extract compared to vehicle treated ones; The cells were reduced in size and abnormally shrunken, compared to other cancer cell lines (PC-3, RCC-45, and MCF7) included in the assay where they showed very small or no difference with their vehicle treated counterparts (Fig. 2). Notably, the extract showed negligible morphological change in normal WI-38 cells (Fig. 2). These results suggested that the toxicity of the extract is highly specific to colon cancer cells with non-toxicity to normal cells at a specific concentration.

### Effect of *Eclipta alba* methanol extract on colony formation of HCT-116 cell

**Fig. 3:**
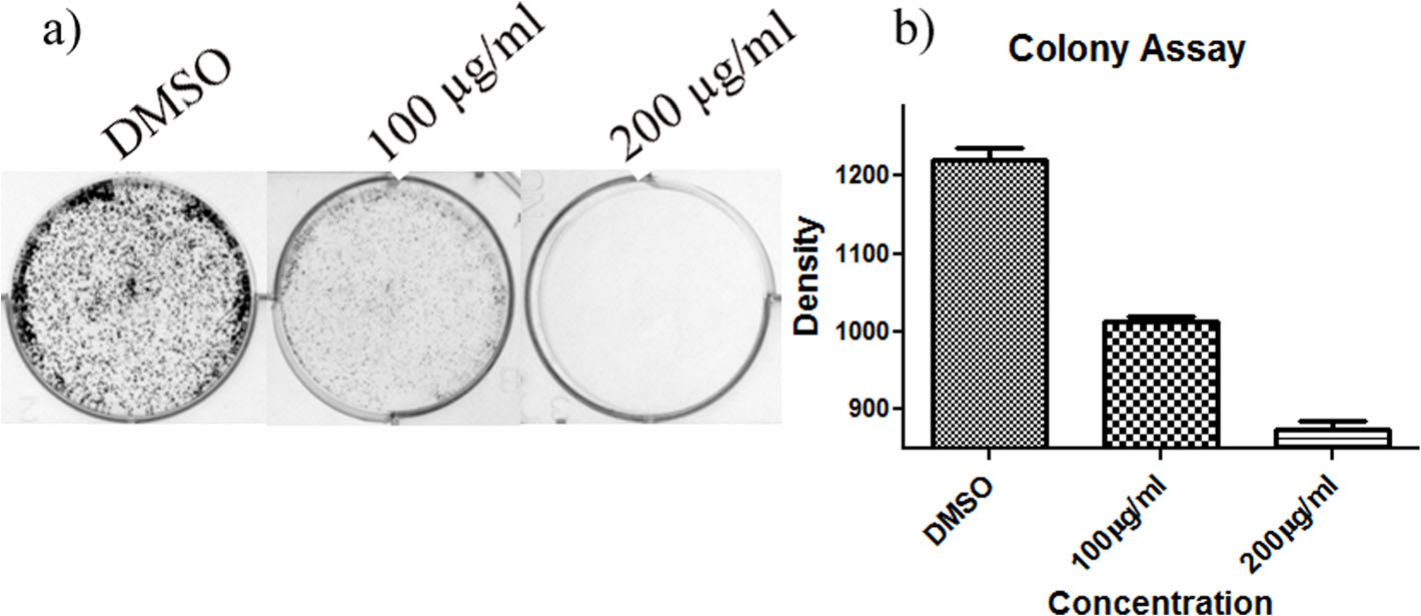
**a** *Eclipta alba* methanol extract (EAME) Inhibit colony formation of HCT-116 cells at the indicted concentrations compared to the control group. **b** Bar graph representing the density of colonies shown in panel **a**

To further investigate anticancer potential of *Eclipta alba* on HCT-116 cells, colony formation assays were carried out. Generally the cancer cells have a tendency to grow in colonies in contact with the neighboring cells; losing the connection with the adjacent cells, results in the death of the cancer cells. Clonogenic assay reveals that treating the cells with the extract, significantly inhibited the colony formation of HCT-116 cells compared to the vehicle (DMSO) group, (Fig. 3). The colonial/population density in each plate after the treatment was measured using Gel Doc and plotted. The density measurement revealed that the plant extract at 100 μg/ml (almost half the dose of IC-50) reduced the growth of colonies by more than 50% compared to control, while treating with the extract at 200 μg/ml (a dose closer to IC-50) decreased the colony formation by more than 90% in comparison with the control group. This assay further conformed the anticancer effect of the *Eclipta alba* extract.

### Effect of *Eclipta alba* methanol extract on migration of HCT-116 cells

**Fig. 4:**
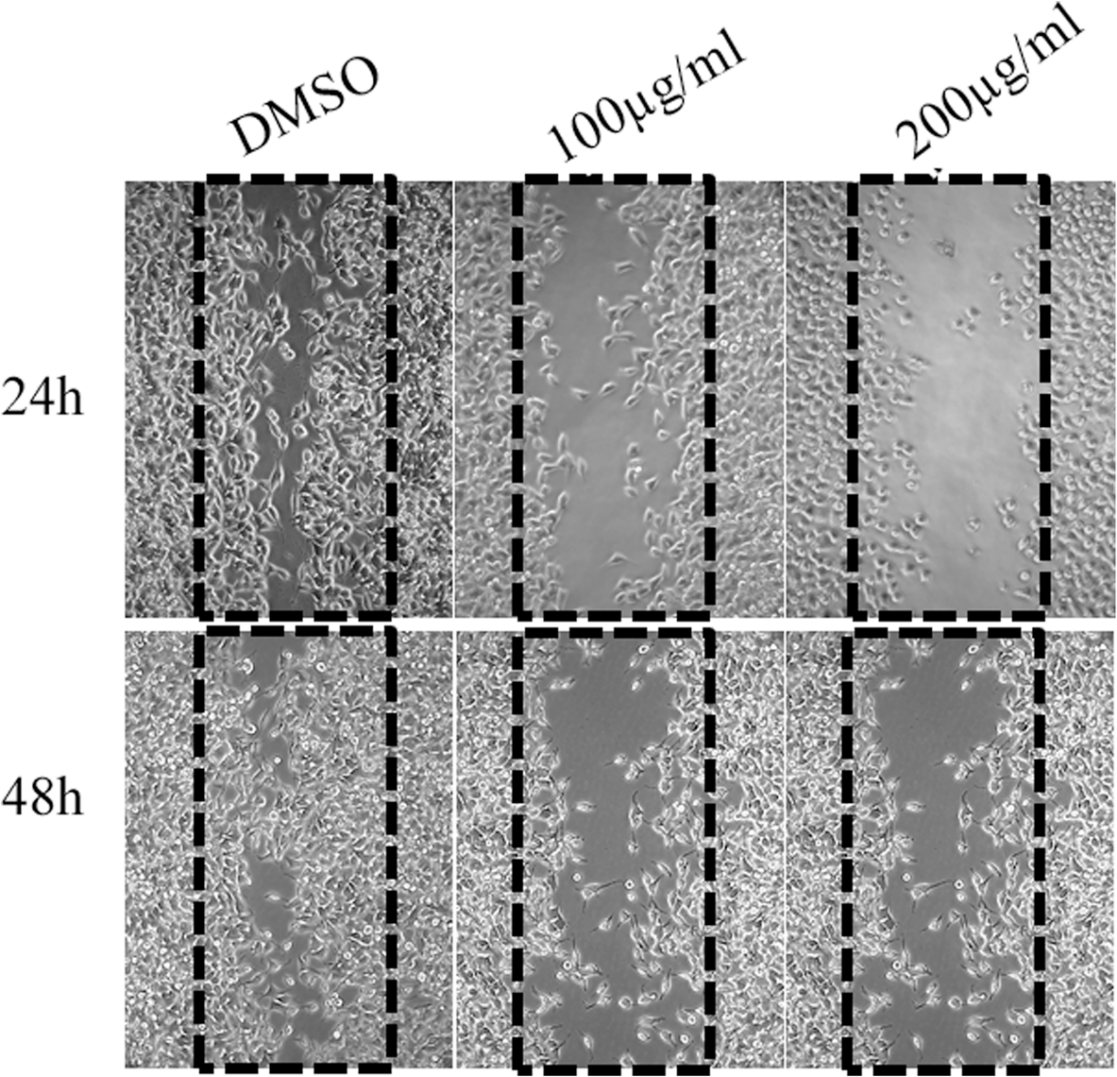
*Eclipta alba* methanol extract inhibits migration of HCT-116 cells: Phase contrast images of cells after treatment with the vehicle (DMSO) or indicated doses of the extract as described in Methods

Wound healing assay was conducted to determine the effect of plant extract on migration capability of HCT-116 cells. The cells were seeded in 6-well plate and treated with a fixed concentration of EAME for 24 h and 48 h. the migration ability of cells were measured under inverted phase contrast microscope. The results of the experiment (Fig. 4) reveals that, there was significant inhibition of wound healing or migration in EAME treated groups in tested concentrations (100 and 200 μg/ml) compared to vehicle treated group as there was no inhibition of wound healing, and the gap was almost covered in 48 h time. When HCT-116 cells were treated with100 μg/ml, a concentration close to about half of IC-50 value of the extract, for 24 h a clear inhibition of the migration of cells was noticed with no visible change in their morphology. However, a clear change in morphology of cells was noticed after 48 h of incubation in addition to a significant inhibition of their wound healing function. A clear morphological change along with inhibition of the migration in HCT-116 cells was observed even at 24 h incubation (and at 48 h, as expected) when treated with 200 μg/ml (IC50 179 ± 0.81 μg/ml) (Fig. 3). As expected, the sensitive cells started dying. These results again support the conclusion that this extract (EAME) has potential anticancer property against HCT-116 cells.

## Discussion

The incidence of colon cancer has been increasing constantly because of the recent changes in life style which include consumption of foods with low/no vegetables and fruits, lack of physical activities/exercise, consumption of excess alcohol and exposure to hazardous chemical substances [19, 20]. Although routine check-up and early detection has reduced the death rate, yet colon cancer claims a large number of lives every year globally. Therefore, there is an urgent need for identifying novel therapeutics or drug candidates which will specifically act on the cancer cells without affecting the normal cells [21].

Here, we explored the anticancer activity of *Eclipta alba* methanol extract EAME against various human cancer cells such as HCT-116, PC-3, MCF-7 and RCC-45. WI-38 cells line was chosen to evaluate the effect of the extract on normal cells. These cells were selected based on the availability as well as on their sensitivity. Initial screening of anticancer activity of EAME was done on HCT-116, HT-29, PC-3, MCF-7, RCC-45 and H-1299 (lung cancer cell lines). Based on their sensitivities to this extract HCT-116, PC-3, MCF-7 and RCC-45 cells were then chosen for further studies. HCT-116 was more sensitive compared to HT-29 (may be due to presence of more mutated oncogenes in HT-29 compared to HCT-116), we decided to test with HCT-116 colon cancer cells and so did not continue with HT-29. More over as revealed here, the EAME showed very specific toxicity towards colon cancer cells HCT-116 compared to the other cancer cells PC-3, MCF-7 and RCC-45 cells (Fig. 1). Notably, the toxicity of EAME to the colon cancer cells HCT-116 was more appreciated after identifying its relative nontoxicity on normal cells WI-38 based on all three types of assays used here (Figs. 1 & 2).

Cancer cells possess higher basal level of reactive oxygen species (ROS) because of its higher metabolic rate and other functions which is distinct from normal cells [22]. Elevated level of ROS is essential for cancer cells to grow, proliferate as well as for metastasis. At the same time an excess over the required level of ROS may lead cancer cells to oxidative stress and possible death [23]. Cancer cells in contrast to the normal cells were shown to carry limited antioxidant mechanism required to scavenge the excess ROS produced, and to prevent associated cellular damage [23]. It is possible that anticancer activity of the extract studied here is due to the presence of compounds that altered the redox balance essential for the survival of the HCT-116 cells. This activity may be either inducing ROS level or inhibiting the ROS level in HCT-116 cells [23]. Negligible toxicity of EAME towards the normal cell is probably due to the presence of strong antioxidant and anti-inflammatory mechanisms present in the normal cells [24]. β-sitosterol, a phytosterol isolated from methanolic extract of *Eclipta alba* was reported to possess antioxidant as well anticancer property, and induce apoptosis via caspase 8-dependent pathways in cancer cells [25]. The antioxidant mechanism shown by the other compounds i.e., wedelolactone, oleanolic acid and eclalbasaponins present in the EAME might be responsible for the observed activity against HCT-116 cells and negligible toxicity towards the normal cells. Further investigations on these compounds will be necessary to understand exact mechanism of specificity towards cancer cells in contrast to the normal cells. The IC-50 values of HCT-116, MCF-7, PC-3 and RCC-45, were found to be 179 ± 0.81, 400 ± 1.01, 470 ± 1.04 and 498 ± 1.90 μg/ml, respectively suggesting relative HCT-116 cell specific effect of *Eclipta alba* extract. The selectivity index (SI) (data not included) obtained from IC-50 values suggested the level of specificity towards the cells. The mechanism underling the cell specific toxicity induced by the extract may be the result of DNA damage as well. Although in general uncontrolled cellular growth due to change in multiple cellular pathways results in cellular transformation, yet different cancers are unique in their own way due to harboring their unique set of mutations [26]. In fact, many cancers carry their individual markers. Colon cancer was shown to be the results of chromosomal instability resulting in the activation of proto-oncogene KRAS. In addition, colorectal cancer also carry mutation in multiple tumor suppressors such as loss of p53, adenomatous polyposis coli (APC) and the defect in the long arm of chromosome 18 due to loss of heterozygosity (LOH) [27]. HCT-116 cells carry KRAS mutation/activation. In many cases breast cancer is caused by mutation in BRCA1 and BRCA2 genes [28]. Therefore, relatively higher sensitivity of HCT-116 cells to this extract is the reflection of its unique genetic nature. Additional experimentation will be required to obtain a deeper insight into the cellular process targeted by the extract.

This study revealed that the extract efficiently blocked two essential properties required by cancer cells for their growth and proliferation, that is, the ability of cancer cells to grow and proliferate in forming colony and migrate for metastasis. The extract could block both of these essential properties of HCT-116 cells. In cancer cells metastasis is a complex biological process that includes invasion and migration of cancer cells; it is also a major cause of death in cancer patients. Currently, many independent groups are engaged to find suitable drug candidates to inhibit metastasis to control the growth of tumor [29]. The results presented here provided dependable evidence that the methanolic extract of *Eclipta alba* carries promising anti-colon cancer compounds which would be worth following-up in the future to understand and for possible therapeutic development.

## Conclusion

The present study concludes that, the methanolic extract of *Eclipta alba* carries potent activity specifically against colorectal cancer cells HCT-116, with ideal characteristics such as with minimal toxicity towards normal cells WI-38. The results also revealed that the extract also significantly inhibited the colony formation and also the migration property of cancer cells in a dose-dependent manner. This unique mechanism can qualify the *Eclipta alba* extract to become a better treatment option for colon cancer patients as appropriate.

## Data Availability

The datasets used and/or analyzed during the current study is available from the corresponding author on request.

## Abbreviations

DMEM: Dulbecco’s Modified Eagle’s Medium
EAME: *Eclipta alba* methanol extract
